# Synchrotron-Radiation-Based Fourier Transform Infrared Microspectroscopy as a Tool for the Differentiation between Staphylococcal Small Colony Variants

**DOI:** 10.3390/antibiotics11111607

**Published:** 2022-11-11

**Authors:** Amal G. Al-Bakri, Lina A. Dahabiyeh, Enam Khalil, Deema Jaber, Gihan Kamel, Nina Schleimer, Christian Kohler, Karsten Becker

**Affiliations:** 1 Department of Pharmaceutics and Pharmaceutical Technology, School of Pharmacy, The University of Jordan, Amman 11942, Jordan; 2 Department of Pharmaceutical Sciences, School of Pharmacy, The University of Jordan, Amman 11942, Jordan; 3 Department of Clinical Pharmacy, School of Pharmacy, Zarqa University, Zarqa 13110, Jordan; 4 SESAME Synchrotron (Synchrotron-Light for Experimental Science and Applications in the Middle East), Allan 19252, Jordan; 5 Department of Physics, Faculty of Science, Helwan University, Cairo 11792, Egypt; 6 Institute of Medical Microbiology, University Hospital Münster, 48149 Münster, Germany; 7 Friedrich Loeffler-Institute of Medical Microbiology, University Medicine Greifswald, 17475 Greifswald, Germany

**Keywords:** staphylococci, small colony variant, synchrotron, FTIR microspectroscopy, *S. epidermidis*, PCA

## Abstract

Small colony variants (SCVs) are clinically significant and linked to persistent infections. In this study, synchrotron-radiation-based Fourier transform infrared (SR-FTIR) is used to investigate the microspectroscopic differences between the SCVs of *Staphylococcus aureus* (*S. aureus*) and diabetic foot *Staphylococcus epidermidis* (*S. epidermidis*) in two main IR spectral regions: (3050–2800 cm^−1^), corresponding to the distribution of lipids, and (1855–1500 cm^−1^), corresponding to the distribution of protein amide I and amide II and carbonyl vibrations. SR-FTIR successfully discriminated between the two staphylococcal species and between the SCV and the non-SCV strains within the two IR spectral regions. Combined *S. aureus* SCVs (SCVhMu) showed a higher protein content relative to the non-SCV wild type. Complemented *S. aureus* SCV showed distinguishable differences from the SCVhMu and the wild type, including a higher content of unsaturated fatty acids. An increase in the CH_2_/CH_3_ ratio was detected in *S. epidermidis* SCV samples compared to the standard control. Protein secondary structure in standard *S. epidermidis* and SCVs consisted mainly of an α-helix; however, a new shoulder at 1635 cm^−1^, assigned to β-sheets, was evident in the SCV. In conclusion, SR-FTIR is a powerful method that can discriminate between staphylococci species and to differentiate between SCVs and their corresponding natural strains.

## 1. Introduction

As an adaptation to an intracellular lifestyle, small colony variants (SCVs) represent a slow-growing subpopulation with unusual metabolic features influencing their phenotype, pathogenic characteristics and persistence [1,2,3]. As main features, they show pinpoint colonies and unusual metabolic auxotrophies [4]. Infections caused by SCVs are clinically significant and challenging due to being chronic, recurrent and antibiotic-resistant [2,3,5,6,7,8]. Persistent and relapsing infections attributed to SCVs have also been reported for diabetic foot ulcers (DFUs) [9]. The SCV phenotype has firstly and mostly been described among *Staphylococcus aureus* (*S. aureus*). However, other staphylococcal species and a range of species of other genera exhibit this phenotype [3,10,11,12,13,14,15].

Transcriptomic, proteomic and metabolomic studies on the SCVs have revealed considerable differences between the wild-type and SCV phenotypes, especially in the fermentative pathways [16,17,18,19,20]. Nevertheless, little is known about the effect of adapting this phenotype to the bacterial biochemical fingerprint [21], and there is a gap in knowledge of the characteristics of this phenotype and its genesis across different microorganisms [3].

Conventional Fourier transform infrared (FTIR) spectroscopy is a non-invasive technique that has been used to successfully describe and type bacteria at various taxonomic levels [22,23,24]. It collects complex biochemical-fingerprint-like spectra derived from the total cell lipid and protein profiles without destroying their biological structures. FTIR spectral analysis was used as a spectroscopic tool for *S. aureus* subtyping and was shown to have a discriminatory power similar to *spa* typing and pulsed-field gel electrophoresis (PFGE) [25]. This technique was also found to be a rapid and reproducible tool able to differentiate between the SCV phenotype and the normal phenotype (NP) where SCVs exhibited a distinguishable FTIR fingerprint from their parent strains [21].

Synchrotron-radiation-based Fourier transform infrared (SR-FTIR) microspectroscopy represents an advancement over conventional FTIR spectroscopy because it gives a better signal/noise ratio at the highest spatial resolution due to the high brightness and collimation of synchrotron radiation [26]. SR-FTIR microspectroscopy is a promising analytical tool that has received increasing interest in recent years for the analysis of biochemical components of biological samples from different matrices. The high spatial resolution of the synchrotron IR source permits subcellular chemical mapping among a range of biological samples [27,28,29,30]. Moreover, the SR-FTIR method was proved to be a specific, reliable technique that can be used to differentiate between different types of bacteria and to analyze bacterial and fungal biofilms [31,32]. The use of this sensitive microspectroscopy technique in studying SCVs might help in identifying spectroscopic differences between the SCVs of different species in addition to differences in the spectroscopic features among the SCV and the NP.

Naumann and Helm (1991) have demonstrated that each microorganism has highly specific IR absorption signatures, correlates with genetic information and provides a specific fingerprint of its nucleic acid, protein, lipid and carbohydrate composition [23,33]. Originally, Naumann and Helm [23] determined five typical bacterial absorption spectral windows (cm^−1^): (i) lipids (3000–2800 cm^−1^ dominated by vibrations of functional groups usually present in fatty acids); (ii) proteins and peptides (1700–1500 cm^−1^, dominated by vibrations of amide I and amide II bands); (iii) a mixed region (1500–1200 cm^−1^, with information of proteins, fatty acids and phosphate-carrying compounds); (iv) polysaccharides (1200–900 cm^−1^, dominated by absorption bands of the carbohydrates present within the cell wall); and (v) a fingerprint region (900–700 cm^−1^, showing some remarkably specific spectral patterns, not yet assigned to cellular components or functional groups). Moreover, it was demonstrated that wavelength absorption regions 1500–1200 cm^−1^ and 1200–900 cm^−1^ are consistently shown to be the most discriminatory and powerful regions for bacterial identification, as reviewed by Novais et al. [34].

The purpose of this study was to evaluate the discriminatory power of SR-FTIR for accurate differentiation between normal and SCV phenotypes of *S. aureus* and *S. epidermidis* applying *S. epidermidis* SCV isolates recovered for the first time in this study, from DFU lesions and a *S. aureus* strain set comprising natural and genetically defined normal and SCV phenotypes.

## 2. Results

### 2.1. Biochemical Characteristics of S. epidermidis SCV Strains 

*S. epidermidis* DFU clinical isolates morphological and biochemical characteristics are described in Table S1. 

### 2.2. IR Spectral Features, Peak Assignments and Group Comparison

**Figure 1:**
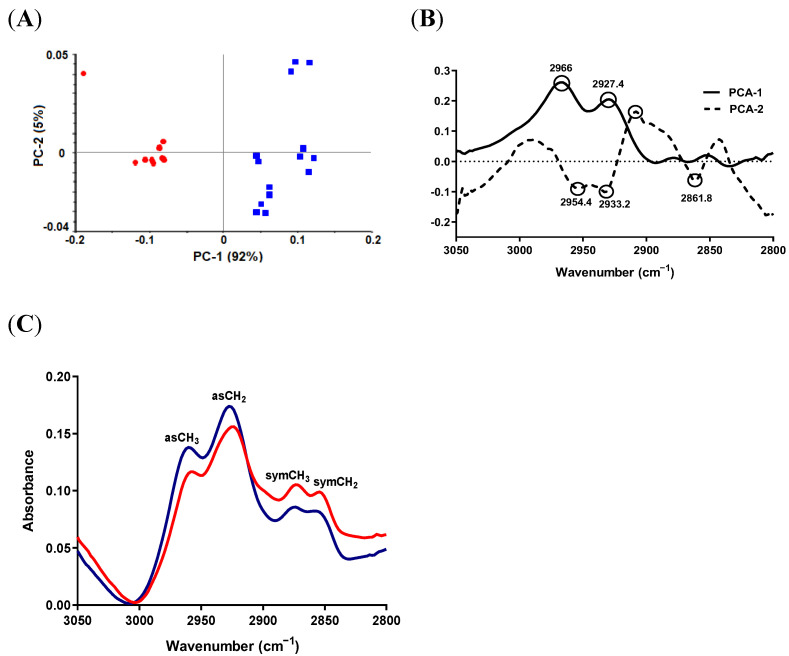
SR-FTIR spectra of lipid region from *S. aureus* ATCC 29213 (blue) and *S. epidermidis* ATCC 12228 (red). (**A**) PCA score plot. (**B**) Loading plots of PC-1 and PC-2 of PCA models of lipid region. (**C**) Unit-vector-normalized average raw spectra of lipid peaks and unsaturated lipid region.

**Figure 2:**
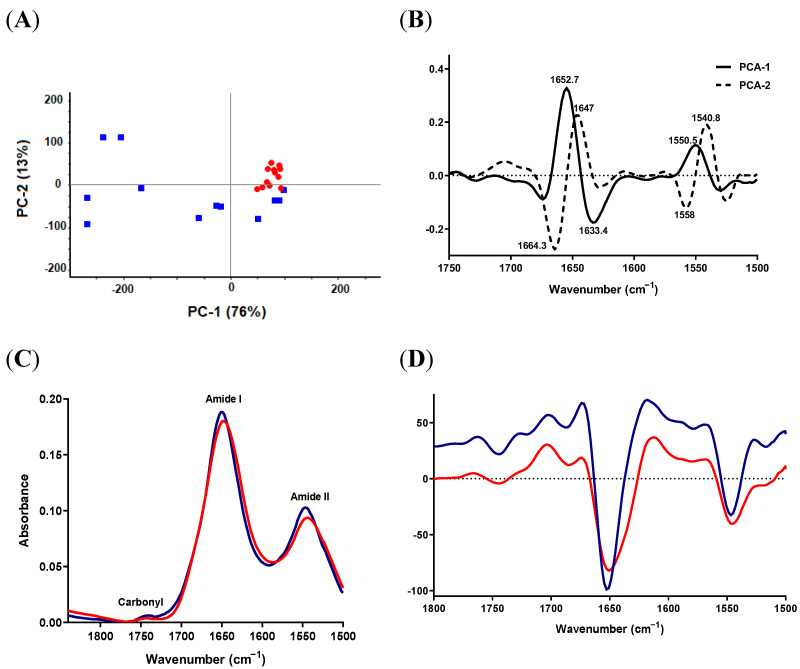
SR-FTIR spectra of protein-carbonyl region from *S. aureus* ATCC 29213 (blue) and *S. epidermidis* ATCC 12228 (red). (**A**) PCA score plots. (**B**) Loading plots of PC-1 and PC-2 of PCA models (**C**) Unit-vector-normalized average raw spectra. (**D**) Normalized reduced Savitzky–Golay second-derivative average spectra of protein-carbonyl region.

**Figure 3:**
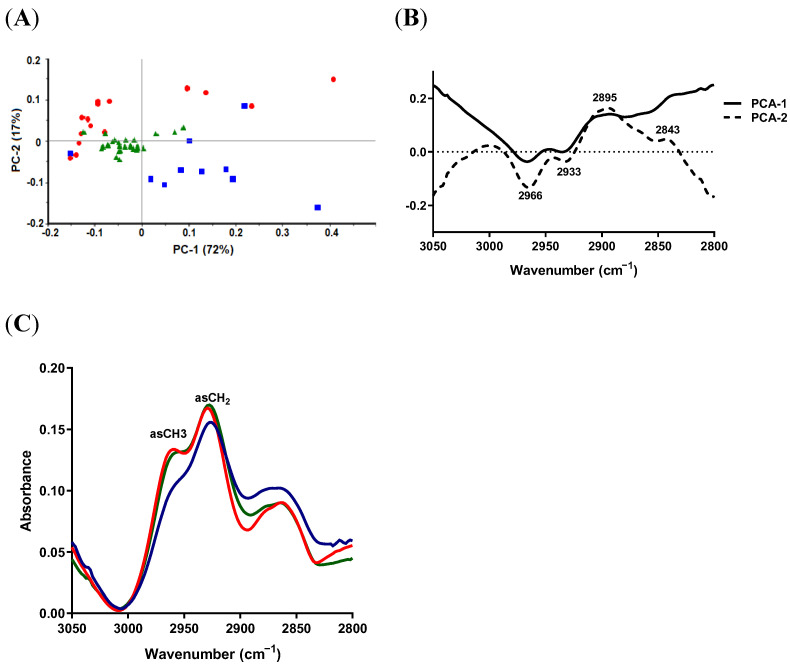
SR-FTIR spectra of lipid region from *S. aureus* wild type (SaW; red), combined natural SCV and *hemB* mutant (SCVhMu, green) and complemented mutant (SaCMu; blue). (**A**) PCA score plot. (**B**) Loading plot of PC-1 and PC-2 of PCA model. (**C**) Unit-vector-normalized average raw spectra.

**Figure 4:**
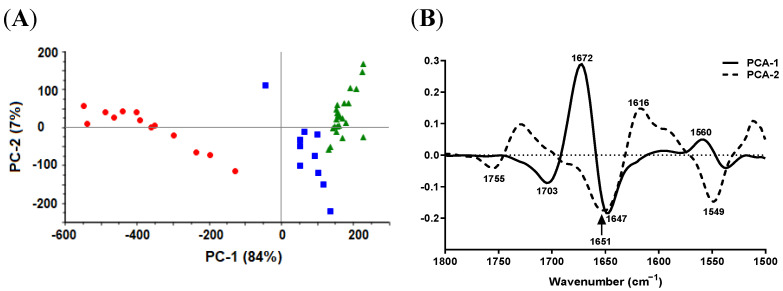

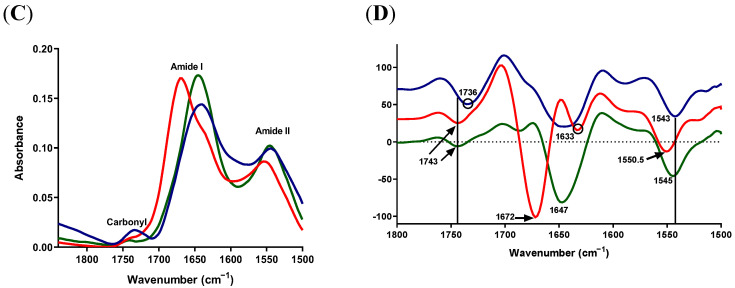
SR-FTIR spectra of protein-carbonyl region from *S. aureus* wild type (SaW; red), combined natural SCV and *hemB* mutant (SCVhMu, green) and complemented mutant (SaCMu, blue). (**A**) PCA score plot. (**B**) Loading plot of PC-1 and PC-2 of PCA model. (**C**) Unit-vector-normalized average raw spectra. (**D**) Normalized reduced Savitzky–Golay second-derivative average spectra.

**Figure 5:**
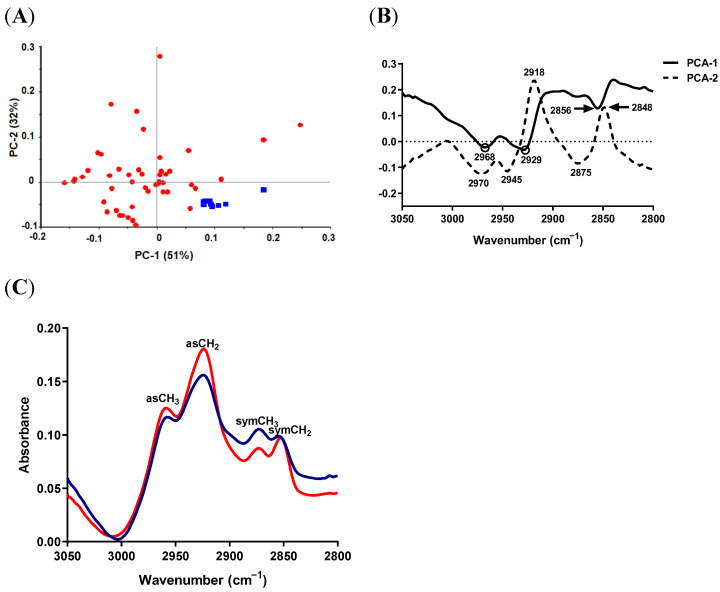
SR-FTIR spectra of the average lipid region from *S. epidermidis* SCV (NSCV, ASCV and LSCV; red) and *S. epidermidis* ATCC 12228 (blue). (**A**) PCA score plot. (**B**) Loading plot of PC-1 and PC-2 of PCA model. (**C**) Unit-vector-normalized average raw spectra of lipid peaks and unsaturated lipid region.

**Figure 6:**
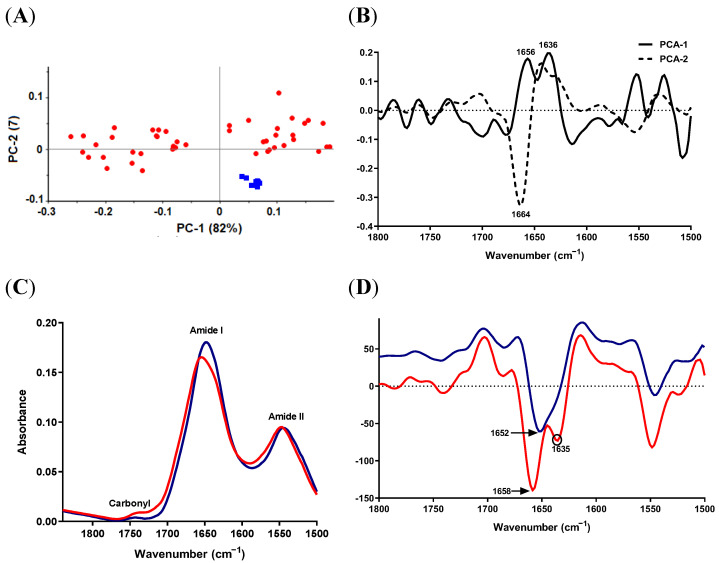
SR-FTIR spectra of the average protein-carbonyl region from *S. epidermidis* SCV (NSCV, ASCV and LSCV; red) and standard *S. epidermidis* (blue). (**A**) PCA scores plot. (**B**) Loading plot of PC-1 and PC-2 of PCA model. (**C**) Unit-vector-normalized average raw spectra. (**D**) Normalized reduced Savitzky–Golay second-derivative average spectra.

Two main IR spectral regions were defined and investigated. The first region (3050–2800 cm^−1^) corresponds to the distribution of lipids, while the second region (1855–1485 cm^−1^) corresponds to the distribution of protein amide I (1700–1600 cm^−1^) and amide II (1600–1485 cm^−1^) and carbonyl vibrations (1855–1700 cm^−1^) [35]. Spectral biochemical features in the two defined regions were compared between the two ATCC strains, *S. aureus* and *S. epidermidis*, as shown in Figure 1 and Figure 2. Additionally, spectral feature differences in the lipid and protein regions between the different *S. aureus* isolates (wild type, natural SCV, *hemB* mutant and complemented mutant) and between the clinical SCV isolates of *S. epidermidis* were identified, as presented in Figure 3, Figure 4, Figure 5 and Figure 6. It is worth mentioning that the PCA score plots in the lipid and protein-carbonyl regions in the IR spectra of the four different *S. aureus* isolates showed an overlap between the IR spectra of the natural SCV and the *hemB* mutant in both regions (Figure S1), reflecting similar biochemical compositions. Therefore, spectra from the natural SCV and the *hemB* mutant were combined for subsequent analysis and were referred to as SCVhMu. Wild-type and complemented mutant groups were separated from each other and from the overlapped *hemB* mutant and natural SCV groups (Figure S1) and subsequently were considered separate groups. Table 1 summarizes position assignments of lipids and amide I and amide II component bands altered in SCVs of *S. aureus* and *S. epidermidis*.

### 2.3. Lipid and Protein Alterations between Reference Strains of S. aureus and S. epidermidis 

#### 2.3.1. Lipid Region

Evident and clear separation was noticed between *S. aureus* and *S. epidermidis* spectra in the PCA score plot, which describes in total 97% of the variability in the data (Figure 1A). The score plot showed that *S. aureus* spectra fell within the positive space of PC1, whereas the spectra of *S. epidermidis* were within the negative space of PC1. The loading of PC1 and PC2 of the PCA model revealed that the most important discriminative variables for PC1 were 2966 and 2927.4, corresponding to asCH_3_ and asCH_2_ stretching vibrations, respectively (Figure 1B). 

When compared to *S. epidermidis, S. aureus* exhibited an increase in the absorption signal intensity in the regions of asCH_3_ and asCH_2_ and a decrease in the signal intensity of symCH_3_ and symCH_2_ peaks, indicating a significant change (difference) in the lipid profile (Figure 1C). Besides the changes in peak intensity, the asCH_2_ peak (assigned to long-chain fatty acids and phospholipids [36]) shifted to a lower wavenumber in *S. epidermidis* (2923.6 cm^−1^) compared to *S. aureus* (2927.4 cm^−1^), indicating possible differences in lipid composition.

**Table 1:** Position assignments of lipids, amide I and amide II component bands altered in SCVs of *S. aureus* and *S. epidermidis*.

Peak Wavenumber (cm^−1^)	Structure	Alterations
2958.3	Asymmetric CH_3_: cholesterol esters, triglycerides	Shift to higher frequency (2958.2 cm^−1^ in SaW → 2960.2 cm^−1^ in SaCMu and SCVhMu)
2927.4	Asymmetric CH_2_: long-chain fatty acids, phospholipids	Shift to lower frequency (2927.4 cm^−1^ in *S. aureus* → 2923.6 cm^−1^ in *S. epidermidis*). Shift to higher frequency (2927.4 cm^−1^ in SaW → 2929.4 cm^−1^ in SaCMu and SCVhMu).
1652.7	Amide I, α-helix	Shift to lower frequency (1652.7 cm^−1^ in *S. aureus* → 1650.7 cm^−1^ in *S. epidermidis*)
1639	Amide I, β-sheets	Main protein secondary structures in SaCMu and SCVhMu
1647	Amide I, random coil	Main protein secondary structures in SaCMu and SCVhMu
1672	Amide I, random coil	Main protein secondary structures in SaW
1743.4	Lipid-carbonyl	Shift to lower frequency (1743 cm^−1^ in SaW and SCVhMu → 1736 cm^−1^ in SaCMu)

Spectral assignments were adapted from references [28,36,37].

#### 2.3.2. Protein-Carbonyl Region

The PCA score plot revealed a good separation of the two species (Figure 2A). Most *S. epidermidis* measurements were clustered in the PC1-positive hemispace of the plane, whereas *S. aureus* was scattered in the score plot but separated from *S. epidermidis*. PC1 and PC2 loading of the second-derivative protein region (Figure 2B) were characterized by four strong peaks in the area of amide I with opposite directions; 1652.7 and 1647 cm^−1^ were positively correlated with PC1 and PC2, respectively, while 1633.4 and 1664.3 cm^−1^ were negatively correlated with PC1 and PC2. Moreover, the loading plot showed medium peaks in the region of amide II pointing to opposite directions, as presented in Figure 2B. The average raw spectra revealed a small decrease in the signal intensity of amide I and amide II peaks in *S. epidermidis*, which might reflect a lower protein content (or synthesis) in *S. epidermidis* compared to *S. aureus* (Figure 2C). With regard to the carbonyl region, no differences could be detected between the two species in the raw spectra (Figure 2C). 

Careful examination of the second-derivative spectra (Figure 2D) showed that the strongest signal in the loading plot and, hence, the most important variable for group separation (1652.7 cm^−1^), shifted from 1652.7 cm^−1^, in the case of *S. aureus*, to a lower wavenumber of 1650.7 cm^−1^ in *S. epidermidis.* This could indicate a conformational change in the protein secondary structure, mainly in the α-helix structure. 

### 2.4. Lipid and Protein Alterations in S. aureus SCV Isolates

#### 2.4.1. Lipid Region

The PCA score plot showed good separation between wild-type (SaW), complemented mutant (SaCMu) and combined natural SCV and *hemB* mutant (SCVhMu) groups and could describe in total 89% of the variability in the data (Figure 3A). The loadings of the PCA model revealed that the most important discriminative variables were 2966 and 2933, corresponding to asCH_3_ and asCH_2_ stretching vibrations, respectively (Figure 3B). Comparison of the average raw spectra identified obvious differences in the absorption signal intensity in all lipid regions of the complemented mutant group compared to the other two groups, which showed minor differences in their lipid profiles (Figure 3C). In the wild-type group, slight blue shifts were noticed with peaks corresponding to asCH_3_ and asCH_2_ when compared to the other two groups, with peaks from 2958.2 to 2960.2 cm^−1^ and from 2927.4 to 2929.4 cm^−1^, respectively.

#### 2.4.2. Protein-Carbonyl Region

Biochemical changes in the protein region between the three groups were more recognizable than those in the lipid region. The PCA score plot clearly showed grouping and separation of the spectra of the three *S. aureus* isolates (Figure 4A). The PCA score plot described in total 95% of the variability in the data; PC1 and PC2 components explained 84% and 7% of the total variance in the spectra, respectively. The PC1 loading of the second-derivative protein-carbonyl region (Figure 4B) was characterized by two strong peaks at 1672 and 1647 cm^−1^ attributed to the amide I peak in the wild type, amide I in SCVhMu and the complemented mutant *S. aureus*, respectively (Figure 4B–D).

The average raw and second-derivative spectra of the three *S. aureus* isolates revealed noticeable differences between the three groups in terms of peak position and intensity, and the appearance of a new shoulder at 1633 cm^−1^ (Figure 4C,D), which might reflect considerable conformational changes and rearrangement in protein secondary structure. A decrease in the amide I absorption signal in the complemented mutant, in comparison to the wild-type and SCVhMu isolates, was noted (Figure 4C). On the other hand, the wild type was associated with a lower amide II absorption signal compared to complemented mutant and SCVhMu isolates (Figure 4C). 

Second-derivative spectra (Figure 4D) indicate that protein secondary structures in the SCV and the complemented mutant consisted mainly of β-sheets (evident at 1639 cm^−1^) and unordered conformation (random coil evident at 1647 cm^−1^). In wild-type *S. aureus*, β-turn structures (evident at 1672 cm^−1^) were the most predominant protein secondary structure, while β-sheets (evident at 1633 cm^−1^) were present with a weaker intensity as presented in Figure 4D.

Of note, the second-derivative spectra (Figure 4D) revealed that the carbonyl stretching peak was more intense in the complemented mutant than the wild type and SCVhMu, and shifted to a lower wavenumber (1743 cm^−1^ in the wild type and SCVhMu to 1736 cm^−1^ in the complemented mutant). 

### 2.5. Lipid and Protein Alterations in S. epidermidis Isolates

The IR spectra of the three clinical isolates obtained from patients were first compared using PCA multivariate analysis to identify any significant differences between the samples in terms of the protein and lipid biochemical composition. PCA score plots in both regions showed an overlap between the groups with no evident separation or clustering (Figure S2). Consequently, the IR spectra of the clinical isolates were combined and compared to standard *S. epidermidis*.

#### 2.5.1. Lipid Region

Clear separation was noticed between SCV samples and the reference *S. epidermidis* in the PCA score plot, which describes in total 83% of the variability in the data (Figure 5A). Reference *S. epidermidis* spectra were clustered within the positive space of the PC1 component. The most important discriminative variables in the PCA loading plot were 2929, 2968 and 2856 cm^−1^ for PC1, and 2918 and 2848 cm^−1^ for PC2 (Figure 5B). The PC1 component was positively correlated with 2856 cm^−1^ (standard spectra) and negatively correlated with 2929, 2968 cm^−1^ (clinical isolate spectra), while the PC2 component was positively correlated with 2918 and 2848 cm^−1^.

When comparing the average raw spectra of the two groups (Figure 5C), an increase in the absorption signal intensity was noticed in clinical isolate samples in the regions of asCH_2_ and asCH_3_, while the peak corresponding to symCH_3_ was decreased, indicating a significant change in the lipid profile. 

#### 2.5.2. Protein-Carbonyl Region

As in the lipid region, reference *S. epidermidis* spectra were clustered within the positive space of the PC1 component of the PCA score plot and were separated from SCV samples (Figure 6A). PC1 in the loading plot was characterized by two signals in the amide I region (1656 and 1636 cm^−1^), whereas PC2 had a strong signal at 1664 cm^−1^, as seen in Figure 6B. 

*S. epidermidis* SCV isolates showed a decrease in the amide I signal intensity and an evident blue shift when compared to the reference *S. epidermidis* (Figure 6C). The second-derivative spectra (Figure 6D) revealed higher signal intensity of the carbonyl stretching peak (at 1740 cm^−1^) in SCV isolates compared to the reference *S. epidermidis*, which reflects higher lipid accumulation (higher oxidation state).

## 3. Discussion

Both *S. aureus* and *S. epidermidis* have been reported among the species most commonly isolated from DFU lesions [38,39,40]. Earlier publications reported the isolation of *S. aureus* SCVs from DFUs, and they were linked to persistent and recurrent infections [9,41]. *S. epidermidis*’s SCV contributions to prosthetic joint devices and bloodstream infections have been reported [33]. However, despite *S. epidermidis*’s growing clinical significance and high prevalence in chronic DFU lesions, no previous studies reported the detection of isolates of this species displaying the SCV phenotype in DFU lesions. Thus, to the best of our knowledge, this study is the first to report the isolation of *S. epidermidis* SCVs from DFU lesion specimens. Whether they can potentially contribute to the chronicity, persistence and treatment failure of the DFU infections warrants further studies.

Attempts to differentiate between *S. aureus* subpopulations showed that FTIR spectral analysis resulted in a high discriminatory power similar to that obtained by *spa* typing and PFGE for *S. aureus* clonal complexes using the spectral region 1200–800 cm^−1^ [25]. FTIR was found to discriminate between communities (mostly USA300 and USA400) using first-derivative normalized data from a single narrow spectral region (1361–1236 cm^−1^), which reflects differences in protein amide III and nucleic acid phosphodiester contents. Moreover, FTIR showed reliable discrimination power based on the capsular structure of *S. aureus*. This was through (1200–900 cm^−1^) and (900– 800 cm^−1^) regions (reviewed by [34]).

Recently, Guliev et al. [42] built a predictive model to discriminate *S. aureus* strains from *S. epidermidis* and other pathogens using FTIR spectroscopy. They have found the polysaccharides window (1200−900 cm^−1^) to be the most important spectral region for successful discrimination. The peaks at ∼1050 cm^−1^, ∼1080 cm^−1^ and ∼1150 cm^−1^ are of particular interest. The ratio between peaks at 1080 and 1050 cm^−1^ turned out to be a good discriminating factor between *S. aureus* and *S. epidermidis*. 

Previously, it was reported that the first-derivative infrared spectra are useful for hierarchical clustering of the *S. aureus* SCV and the wild-type phenotype and showed a clear discrimination between both phenotypes [21]. Nevertheless, a clear discrimination between such related organisms, such as the wild-type and SCV phenotypes, needs a powerful tool that can detect small phenotypic differences between these variants where DNA-based methods would usually fail. Herein, for the first time, we report an analysis of the IR spectra of the SCV phenotype of *S. aureus* and *S. epidermidis* using SR-FTIR. In our study, SR-FTIR was a powerful tool for discriminating between the two staphylococcal species within the two main IR spectral regions: (3050–2800 cm^−1^). which corresponds to the distribution of lipids, and (1855–1500 cm^−1^), which corresponds to the distribution of protein amide I and amide II and carbonyl vibrations. SR-IR demonstrated a distinctive lipid profile and composition among *S. aureus* and *S. epidermidis*. Moreover, differences in the protein-carbonyl region showed a lower protein content in *S. epidermidis* and conformational changes in the protein secondary structure mainly in the α-helix compared to that of *S. aureus*. Moreover, the two IR spectral regions, (3050–2800 cm^−1^) and (1855–1485 cm^−1^), were able to discriminate the wild-type from the SCV and the SCVs from the respective reference strains.

The amid I and II results reveal that the *S. aureus* SCVhMu was higher in protein content relative to the wild type, reflecting a possible increase in the synthesis of the most abundant proteins. 

Interestingly, for the complemented *S. aureus* mutant, although it reverted to the normal colony size phenotype, there were distinguishable differences among this strain and the combined SCV (SCVhMu), with a higher content of unsaturated fatty acid. To estimate lipid unsaturation, the ratio of =C-H (3000–3100 cm^−1^) to CH_2_ (2950–2900 cm^−1^), denoted as the unsaturation index, was calculated [43]. A higher level of unsaturation was noticed in the complemented mutant *S. aureus* (26.7%) compared to the combined SCV (SCVhMu) (17.6%), which might indicate a high content of unsaturated fatty acids. However, this needs to be further validated in a future study. This higher degree of fatty acyl chain unsaturation among the complemented mutant increases the susceptibility of fatty acids to lipid peroxidation with a subsequent effect on membrane integrity and permeability. Moreover, the lipid carbonyl peak was more intense and shifted to a lower frequency in the complemented mutant compared to the wild-type and SCVhMu *S. aureus*. The higher intensity might point to a higher lipid accumulation in the complemented mutant, while the shift in the frequency could reflect an alteration in the intra- and/or intermolecular hydrogen bonding of the phospholipid structure interface with water or other functional groups of molecules. These observations correspond to the results of a previous proteomic approach showing that a natural revertant from a clinical *S. aureus* SCV also retained dominant protein features of the clinical SCV phenotype [20].

The increase in the CH_2_/CH_3_ ratio detected in *S. epidermidis* SCV samples (1.15 in controls to 1.3 in clinical samples) might point to transformation of conformation of lipid tails [44]. CH_3_ and CH_2_ stretching vibrations correspond to cholesterol esters and triglycerides, and long-chain fatty acids and phospholipids, respectively [36]. Given that asCH_2_ and symCH_2_ stretching vibrations provide information about the order of hydrocarbon tails in lipids, an increase in the CH_2_/CH_3_ ratio might suggest conformational changes in the order of lipid acyl chains in *S. epidermidis* SCV samples [44]. Protein secondary structure in standard *S. epidermidis* and clinical isolates (SCV) consisted mainly of an α-helix (evident by peaks centered at 1652 and 1658 cm^−1^, respectively [28,29,30] Figure 6D. However, a new shoulder at 1635 cm^−1^, assigned to β-sheets [28,37], was evident in clinical isolates (SCV), pointing to conformational changes and rearrangement of the protein secondary structure in clinical isolates (SCV).

## 4. Materials and Methods

### 4.1. Bacterial Cultures

DFU samples collected from a previous study were used to isolate staphylococcal SCVs [38]. The identification of *S. epidermidis* isolates displaying the SCV phenotype, pinpoint colonies, recovered from DFUs was performed using conventional laboratory testing, including Gram staining, catalase (Merck, Germany) coagulase (Biolife, Italy) and mannitol fermentation. SCVs were identified as *S. epidermidis* using the RapID One System (Remel, USA) kit according to manufacturer’s recommendations. Confirmation of the species was performed by routine matrix-assisted laser desorption/ionization coupled to time-of-flight mass spectrometry (MALDI-TOF MS) mass procedure applying a MALDI Biotyper smart instrument (Bruker, Germany), and spectra were evaluated with the MALDI Biotyper software (Bruker) usually used for standard microbial identification. To exclude *S. aureus*, PCR tests designed for the detection of the *nuc* [45] and *tuf* genes [46] were additionally performed. 

Three *S. epidermidis* strains displaying the SCV phenotype, recovered from DFUs, were included in the study (Table 2). These strains exhibited the SCV phenotype on blood agar after 24–72 hr of incubation. The pinpoint colonies were re-cultured on Colombia agar (Oxoid, UK) and nutrient agar (Oxoid, UK). The stability of the SCV phenotype was tested by subculturing on Colombia agar for at least 15 times.

Auxotrophy testing for *S. epidermidis* SCVs was performed as described elsewhere [33] using 10 μL of hemin (1 mg/mL), menadione (160 μg/mL) and thymidine (100 μg/mL) (Sigma-Aldrich, Inc. St. Louis, MO, USA) on Mueller-Hinton agar (MHA, Merck KGaA, Darmstadt, Germany).

In addition, a set consisting of (i) previously isolated and characterized *S. aureus* clinical parental isolate (A3878-III) displaying the normal phenotype (wild type), (ii) the corresponding clinical SCV isolate (A3878-I), (iii) the corresponding site-directed knockout *hemB* mutant displaying a stable SCV phenotype and (iv) their complemented mutant were used (Table 2) [16,20,47].

Long-term maintenance of the SCV isolates and the other bacterial strains was performed by freezing the culture, supplemented with 10% glycerol, at −70 °C.

### 4.2. Sample Preparation for SR-FTIR Measurements

SaW, SaSCV, SaMu, SaCMu, NSCV, LSCV and ASCV, *S. aureus* ATCC 29213 and *S. epidermidis* ATCC 12228 were cultured on TSA medium (Oxoid, UK). After 24 h, 3–5 identical separated colonies were suspended in 1,000 µL of sterile distilled water and vortexed for 5 min. The optical density was adjusted for each sample at OD_600_ 0.5, and then the samples were centrifuged at 15,000 rpm for 3 min, the supernatant was discarded, and the pellet was reconstituted with 20 µL of sterile distilled water. Prior to analysis, a volume of 5 µL of the bacterial suspension was spread over transmission IR windows (Crystran, Ltd., Poole, UK) to obtain a homogenous bacterial distribution. Samples were allowed to dry for 30 min in a desiccator purged with dry ultra-pure air available from IR beamline, SESAME synchrotron (Synchrotron light for Experimental Science and Applications in the Middle East, Allan, Jordan) 

### 4.3. Synchrotron–Fourier Transform Infrared (SR-FTIR) Microscopic Measurements

SR-FTIR microscopic measurements were performed at the BM02-IR beamline of SESAME synchrotron. All spectra were acquired in transmission mode using a Thermo Nicolet Continuum IR-Vis microscope coupled to 8700 Thermo Nicolet FTIR and Atlµs software (Thermo Fisher Scientific©, Waltham, MA, USA). For each sample, 15-point maps were collected in the mid-infrared range (4000 and 650 cm^−1^) with 4 cm^−1^ spectral resolution and 256 co-added scans. The point maps of bacterial film were acquired using the 15x Schwarzschild objective and a matching 15x condenser.

### 4.4. SR-FTIR Data Treatment and Statistical Analysis

The collected raw spectra were processed using the multivariate statistical analysis software the Unscrambler X 10.4 (CAMO Analytics, Norway), which was used as previously described [48,49,50]. Briefly, the absorption spectra in the two identified spectral regions, lipid CH stretching (2800–3050 cm^−1^) and the protein (amide I and II)-carbonyl vibrations (1485–1855 cm^−1^), were baseline-corrected and smoothed with a 7-point window using the Savitzky–Golay smoothing algorithm (2^nd^ polynomial order). Spectra of the lipid region were unit-vector-normalized while the protein-carbonyl spectral region was differentiated using Savitzky–Golay second derivatives (3^rd^ polynomial order and 7 smoothening points). Finally, the latter spectral region was range-normalized. Principal component analysis (PCA) was performed on each of the two predefined spectral ranges, and results were presented as score and loading plots. The normalized reduced average spectrum for each data set was obtained for both lipid and protein-carbonyl regions and used to compare and capture spectral differences. For the protein-carbonyl region, the normalized reduced Savitzky–Golay second-derivative average spectrum was also obtained. 

## 5. Conclusions

SR-FTIR is a powerful method that was able (i) to discriminate between different species of the same bacterial family, as shown here for *S. aureus* and *S. epidermidis*, and (ii) to differentiate between different phenotypes of the same species, as demonstrated for a staphylococcal normal phenotype and its corresponding natural and genetically defined SCV phenotype. Further studies are warranted to show the inter- and intraspecies discriminatory power for other microbial taxa and phenotypes.

## Figures and Tables

**Table 2:** SCV strains and controls used within the study.

Designation	Species and Phenotype	Reference
SaW ^1^	*S. aureus* (wild type)	[33]
SaSCV ^1^	*S. aureus* (natural SCV)	[33]
SaMu ^1^	*S. aureus* (*hemB* mutant)	[21]
SaCMu ^1^	*S. aureus* (complemented mutant)	[21]
ASa2	*S. aureus* (control) ATCC 29213	
ASe1	*S. epidermidis* (control) ATCC 12228	
NSCV	*S. epidermidis* (natural SCV)	Current work
ASCV	*S. epidermidis* (natural SCV)	Current work
LSCV	*S. epidermidis* (natural SCV)	Current work

^1^ Isogenic *S. aureus* strain set of phenotypically different isolates.

## Data Availability

Data will be available upon request from the corresponding author.

## Supplementary Materials

The following supporting information can be downloaded at: https://www.mdpi.com/article/10.3390/antibiotics11111607/s1, Table S1. Biochemical and other characteristics of DFU SCV strains. Figure S1. PCA-score plot of lipids and protein-carbonyl regions in *S. aureus* wild type, natural SCV, *hemB* mutant and complemented mutant. Figure S2. PCA-score plots of lipids and protein-carbonyl regions, of *S. epidermidis* SCV clinical isolates obtained from three different patients; LSCV, NSCV and ASCV.
