# Supplementary material for: Synchrotron-Radiation-Based Fourier Transform Infrared Microspectroscopy as a Tool for the Differentiation between Staphylococcal Small Colony Variants
Source: Antibiotics (Basel). 2022 Nov 11;11(11):1607. doi: 10.3390/antibiotics11111607 (PMC9686486; doi:10.3390/antibiotics11111607)
Supplement: Supplementary file 1 [file antibiotics-11-01607-s001.zip › antibiotics-1955503-supplementary.pdf]

Supplementary Materials

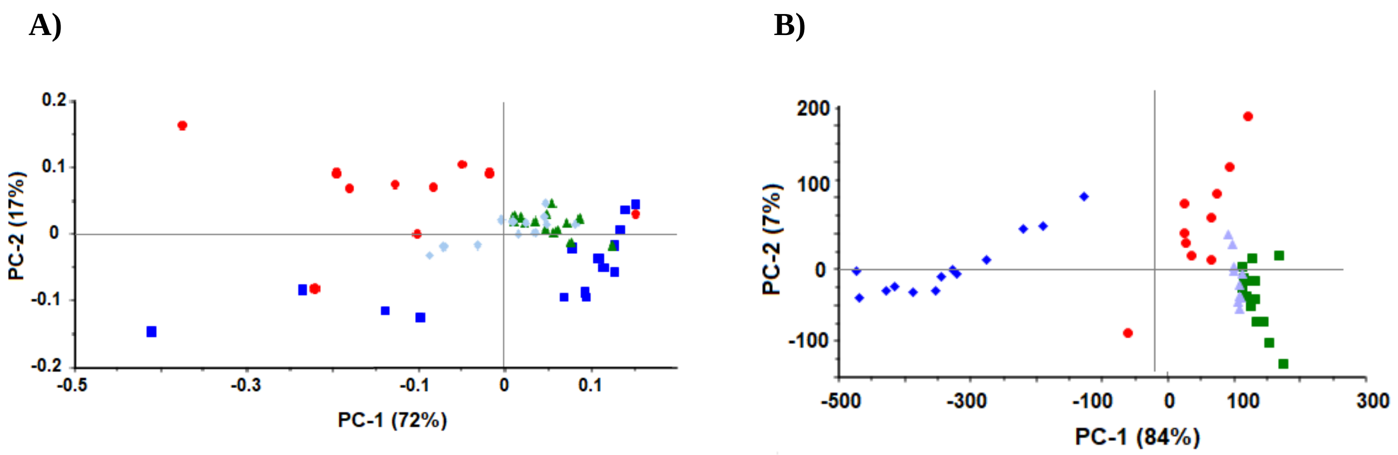

Figure S1: PCA-score plot of lipids **(A)** and **(B)** protein-carbonyl regions in *S. aureus* wild type (navy), natural SCV (green), *hemB* mutant (light blue) and complemented mutant (red).

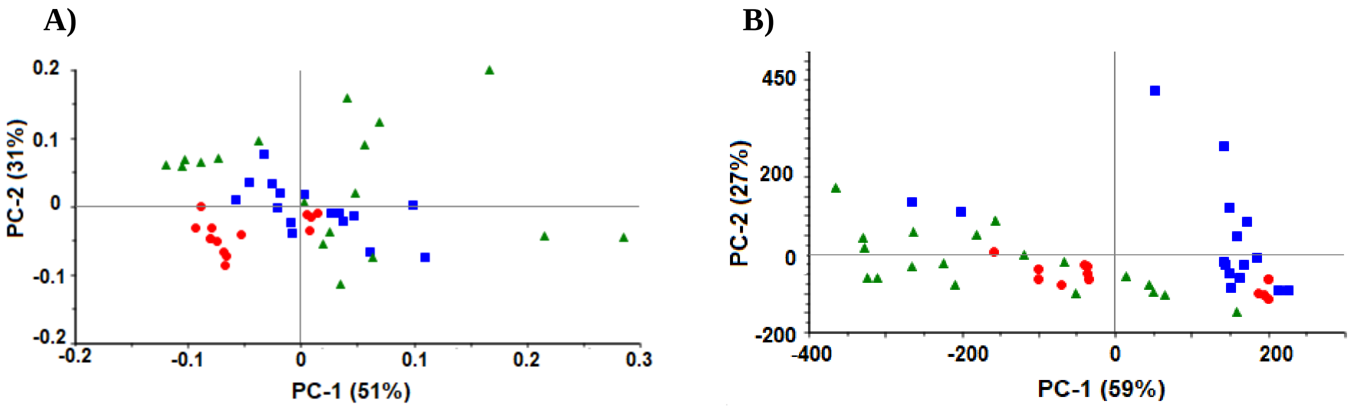

Figure S2: PCA-score plots of lipids **(A)** and protein-carbonyl **(B)** regions, respectively, of *S. epidermidis* SCV clinical isolates obtained from three different patients; red, green and blue (LSCV, NSCV and ASCV, respectively)

Table S1. Biochemical and other characteristics of DFU SCV strains

Sample ID	Growth nutrient agar	Growth Colombia agar	Subculture on Colombia agar for at least 15 times	Gram stain	Catalase	Coagulase	Hemolysis	RapID One System Species	MALDI* (Best Score Value)
**NSCV (DFU clinical isolate)**	SCV	SCV	SCV	Positive	Positive	Negative	Gamma	*Staphylococcus epidermidis*	1.93
**ASCV (DFU clinical isolate)**	SCV	2 types	Converted after 7th culture	Positive	Positive	Negative	Gamma	*Staphylococcus epidermidis*	2.03
**LSCV (DFU clinical isolate)**	SCV	SCV	SCV	Positive	Positive	Negative	Gamma	*Staphylococcus epidermidis*	1.97

*Meaning of score values: 2-3; high confidence identification, 1.7-1.99; low confidence identification, 0-1.69; No organism identification was possible.
